# Construction of a safe and controllable quality management model for “Internet + Nursing Services” based on HFMEA strategy

**DOI:** 10.3389/fmed.2026.1797741

**Published:** 2026-06-03

**Authors:** Lijun She, Lingling Song, Jiajia Tang, Xuemei Wu, Haipin Liang, Xiaohui Qin

**Affiliations:** 1 Department of Nursing, The Third Affiliated Hospital, School of Medicine, Foshan University, Foshan, Guangdong, China; 2 Department of Quality Control, The Third Affiliated Hospital, School of Medicine, Foshan University, Foshan, Guangdong, China; 3 Master of Science in Nursing Class of 2025,Department of Nursing, School of Medicine,Foshan University, Foshan, Guangdong, China; 4 Intensive Care Unit, The Third Affiliated Hospital, School of Medicine, Foshan University, Foshan, Guangdong, China; 5 Department of Urology, The Third Affiliated Hospital, School of Medicine, Foshan University, Foshan, Guangdong, China

**Keywords:** cost-effectiveness, healthcare failure mode and effects analysis (HFMEA), home care, Internet + Nursing Services, nurse satisfaction, patient safety, quality management

## Abstract

**Objective:**

This study aimed to develop and evaluate a healthcare failure mode and effects analysis (HFMEA)-based quality management model for “Internet + Nursing Services” (INS) to enhance service safety, standardization, and stakeholder satisfaction, while assessing its economic impact.

**Methods:**

A retrospective controlled study was conducted. Patients receiving INS from January to December 2022 (*n* = 170) served as the control group, while those receiving services via an HFMEA-optimized platform from January to December 2023 (*n* = 400) constituted the experimental group. Propensity score matching was applied to ensure baseline comparability. The intervention involved a systematic HFMEA process: (1) multidisciplinary team formation and workflow mapping, (2) identification and risk prioritization (via Risk Priority Number, RPN) of potential failure modes across eight key process nodes, (3) implementation of targeted controls (e.g., automated nurse–patient matching, pre-visit checklists, standardized operating procedures, emergency protocols, and a closed-loop reporting system), and (4) integration with a digital platform for workflow management and data tracking. Primary outcomes were the incidence of adverse events and the rate of unplanned re-visits or clinic visits within 48 h. Secondary outcomes included patient and nurse satisfaction scores, mean RPN (process risk), nursing record completion rate, emergency response timeliness, and direct medical costs associated with adverse events.

**Results:**

The HFMEA-based model significantly improved safety and quality metrics. The adverse event incidence was reduced by 76.8% (1.50% in the experimental group vs. 6.47% in the control group, *p* = 0.003), and the unplanned revisit rate decreased from 10.6 to 2.25% (*p* < 0.001). The mean process RPN dropped by approximately 80% (from 172.1 ± 20.8 to 34.6 ± 10.2, *p* < 0.001). Patient satisfaction increased from 92.0 ± 2.7 to 97.2 ± 2.1 (*p* < 0.001), and nurse satisfaction rose from 77.0 ± 13.8 to 90.6 ± 8.4 (*p* < 0.001). Operational efficiency improved, evidenced by a higher nursing record completion rate (98.2% vs. 85.9%) and a 50% reduction in emergency response time (21 vs. 42 min). Structural equation modeling revealed that nurse risk perception mediated 62.5% of the effect of specialized training on patient satisfaction. Economically, the intervention resulted in direct medical cost savings of 16,530 RMB (a 75.6% reduction), averaging 41.3 RMB saved per service case.

**Conclusion:**

The HFMEA-based model was associated with a 76.8% reduction in adverse events, an 80% decrease in RPN, and improved satisfaction. However, causal conclusions are limited by the retrospective, single-center design. Preliminary cost savings were observed, but formal economic evaluation is needed. Replication in diverse settings and future multicenter studies are required.

## 1 Introduction

The integration of digital technologies into healthcare delivery has accelerated globally, giving rise to innovative service models such as ‘Internet + Nursing Services’ (INS). These platforms connect patients requiring skilled nursing care at home with qualified nurses through online applications, addressing growing needs for post-acute care, chronic disease management, and support for aging populations (1, 2). Recent epidemiological and clinical data demonstrate the urgent demand for standardized, safe, and effective home nursing services in digitally mediated care environments (3).

Despite this rapid expansion, significant challenges persist in ensuring the quality and safety of digitally mediated home nursing care. Unlike institutional settings, home environments present unique and variable risks including inconsistent caregiver availability, limited emergency resources, environmental hazards, and communication barriers (4, 5). Compared with traditional hospital-based services, these risks necessitate robust, systematic quality management and safety frameworks, highlighting the importance of integrating HFMEA strategies with digital INS platforms (6). Studies have documented concerns regarding procedural inconsistencies, inadequate risk assessment, documentation gaps, and delayed response to clinical deterioration in existing INS models (7). These safety gaps not only threaten patient outcomes but also contribute to professional anxiety and burnout among nurses working in isolated home settings (8). Furthermore, implementing and scaling INS models face significant technological, organizational, and regulatory barriers, including platform interoperability issues, data privacy and security concerns, unclear legal liability and cross-regional licensure, and the absence of standardized reimbursement policies. These barriers must be addressed alongside clinical risk management to ensure sustainable and safe deployment.

Healthcare failure mode and effects analysis (HFMEA), developed by the U.S. National Center for Patient Safety, offers a structured, proactive approach to identifying and mitigating system failures before they cause harm (9). Originally applied in hospital settings, HFMEA systematically analyzes processes to identify potential failure modes, assess their risks, and implement targeted controls (10). While extensively validated in surgical, medication, and diagnostic contexts, its application to digital home care services remains limited (11). Compared to reactive methods such as Root Cause Analysis (RCA), which investigates incidents after they occur, HFMEA enables prospective risk identification and mitigation. Unlike PDCA cycles that focus on iterative improvement without quantitative risk prioritization, HFMEA provides a structured scoring system (Risk Priority Number) to rank failure modes, offering a more systematic approach for complex, distributed workflows like INS. This represents a significant knowledge gap, given the complex, distributed nature of INS workflows that span digital platforms, transportation, home environments, and follow-up coordination.

Current research on INS predominantly focuses on technological functionality, user acceptance, or satisfaction metrics, with insufficient attention to systematic risk management frameworks (12). Studies applying HFMEA or similar prospective risk assessment methodologies within digitally integrated INS platforms remain limited (13). Furthermore, previous INS research has mostly targeted isolated outcomes, whereas comprehensive evaluations encompassing safety, efficiency, stakeholder satisfaction, and cost-effectiveness simultaneously are still lacking (14). This study addresses these gaps by developing, implementing, and evaluating an HFMEA-based quality management model integrated with a digital INS platform, incorporating structural equation modeling to explore mediating mechanisms and assessing economic outcomes.

The primary objectives are to: (1) systematically identify and prioritize risks in INS workflows using HFMEA methodology; (2) implement targeted interventions based on risk assessment findings; (3) evaluate the impact on adverse event rates, process safety metrics, and stakeholder satisfaction; and (4) assess the economic implications of quality improvement interventions. We hypothesize that the systematic application of HFMEA will significantly enhance INS safety, efficiency, and sustainability while maintaining cost-effectiveness.

This research contributes to the evolving evidence base for digital health quality improvement and provides practical guidance for healthcare organizations implementing technology-enabled home care services. By demonstrating how structured risk management can be integrated with digital platforms, this study offers a replicable model for enhancing patient safety in distributed care environments.

## 2 Materials and methods

### 2.1 Study design and population

This study was a retrospective controlled study. Patients receiving “Internet + Nursing Services” from January 1, 2022, to December 31, 2022, were sequentially selected as the control group. Patients receiving home nursing services via the “Internet + Nursing Service” platform from January 1, 2023, to December 31, 2023, were selected as the experimental group. To minimize intergroup differences arising from the time sequence and potential confounding, propensity score matching (PSM) was employed to construct comparable samples before comparing the control and experimental groups. Covariates in the propensity model included demographic characteristics, underlying diseases, functional status, primary nursing service types, and service year to control for temporal trends and baseline heterogeneity. Residual confounding was further addressed by adjusting for unmatched covariates in subsequent multivariable analyses. The propensity score was established via multivariate logistic regression, incorporating covariates based on the patients’ actual clinical characteristics: (1) Demographic characteristics: Age (continuous variable) and gender. (2) Underlying diseases and health status: Chronic conditions such as diabetes, stroke, and long-term bedridden/debility status (all binary variables), reflecting the patient’s baseline health level and nursing risk. (3) Nursing service-related characteristics: Primary nursing service items, including urethral catheter care, nasogastric tube care, pressure injury dressing change, etc. (coded as binary variables by item category), reflecting the type and complexity of nursing needs. (4) Functional status and care dependency: Whether the patient was long-term bedridden (extracted from “other debilitated long-term bedridden” in Table 1), used to reflect functional status and dependency on nursing care. (5) Service application and appointment method: All patients completed online appointments and physician review through the “Internet + Nursing Service” platform; thus, the operational pathway was consistent and not used as a matching variable. However, “service year” (2022 vs. 2023) was included as a time-trend covariate in the model to adjust for systematic bias potentially introduced by annual policy changes or platform upgrades. Based on the above covariates, the propensity score was calculated. Nearest-neighbor 1:1 matching without replacement was performed, with a caliper set to 0.2 standard deviations of the logit propensity score (caliper = 0.2 × SD of logit). After matching, balance was assessed using standardized mean difference (SMD), with an SMD < 0.1 considered indicative of good balance. The matched sample was used for the primary outcome analysis.

**Table 1:** Comparison of baseline characteristics.

Variable	Control group (*n* = 170)	Experimental group (*n* = 400)	*p*-value
Gender			0.854
Male	62	145	
Female	108	255	
Age (years, mean ± SD)	70.2 ± 12.5	69.6 ± 13.1	0.635
Underlying diseases			
Diabetes	46 (27.1%)	107 (26.8%)	0.944
Stroke	59 (34.7%)	133 (33.3%)	0.751
Other debilitated/long-term bedridden	65 (38.2%)	160 (40.0%)	0.698
Primary nursing service items			
Urinary catheter care	72 (42.4%)	161 (40.3%)	0.66
Nasogastric tube care	88 (51.8%)	212 (53.0%)	0.724
Pressure injury dressing change	44 (25.9%)	106 (26.5%)	

### 2.2 Inclusion and exclusion criteria

Inclusion Criteria: (1) Service application method: Successful registration on the “Internet + Nursing Service” platform, with the service application and appointment process completed independently by the patient or family members under physician guidance. (2) Indications for nursing service: Assessed by a clinical physician as requiring one or more of the following home nursing services, such as indwelling urinary catheter care (including catheter replacement, irrigation, maintenance); nasogastric tube care (including nasogastric tube/gastrostomy replacement, irrigation, patency assessment); pressure injury dressing change (daily dressing and wound care for Stage I–III pressure injuries or stable Stage IV injuries); other common home nursing items, such as PICC line maintenance and tracheostomy care. (3) Stable disease condition: Patients in the chronic disease recovery phase or with long-term bedridden status, showing no signs of acute exacerbation, with relatively stable vital signs, allowing for independent assessment and management by the nurse. (4) Home environment conditions: Home address within the platform’s service coverage area, with a relatively safe environment meeting basic conditions for nurse home visits (e.g., cleanliness, ventilation, presence of a caregiver).

Exclusion Criteria: (1) Unstable condition or requiring emergency treatment: Including, but not limited to, body temperature >38.5 °C with an unclear infectious cause; recent onset of acute/critical manifestations such as impaired consciousness, frequent vomiting, or bleeding. (2) Nursing needs exceeding nurse’s scope of practice: Requiring physician-ordered procedures such as intravenous infusion, emergency resuscitation, or prescription medication adjustments that cannot be performed independently by a nurse. (3) Suspected or confirmed infectious disease patients: Including diseases with significant transmission risk, such as pulmonary tuberculosis or hepatitis B virus in a high replication state. (4) Severe safety hazards in the home environment: Such as the patient residing on a high floor without elevator access, family members exhibiting abnormal behavior (e.g., violent tendencies, severe mental disorders), or situations where personal safety during the service cannot be guaranteed. (5) Inability to communicate or refusal to cooperate: Including patients with severe cognitive impairment without guardian assistance, or patients/family members refusing to cooperate with nurse procedures and assessments.

### 2.3 Methods

Forty-two service nurses participated, including 39 females and 3 males. Eight nurses were under 30 years old, 21 were aged 30–39, and 13 were 40 years or older. Twenty-three nurses had 5 to 10 years of clinical nursing experience, and 19 had over 10 years. Regarding professional titles, 18 were Nurse Practitioners, 21 were Senior Nurse Practitioners, and 3 held titles of Associate Chief Nurse Practitioner or higher.

#### 2.3.1 Control group

Patients in the control group received “Internet + Nursing Services.” Nursing staff provided basic nursing procedures at patients’ homes, including urethral catheter care, nasogastric tube care, pressure injury dressing change, PICC line maintenance, tracheostomy care, and wound dressing. The service process typically involved appointment scheduling via telephone by the patient or family. Nurses made visits based on routine scheduling plans, with no unified information platform for end-to-end service dispatch and supervision. Data for the control group were collected retrospectively from hospital paper records and electronic logs in the Hospital Information System (HIS), ensuring capture of adverse events, nursing procedures, and patient visit information for comparison with the 2023 experimental group. Regarding nursing procedures, urethral catheter care primarily involved catheter replacement, bladder irrigation, and perineal skin care. Nasogastric tube care included replacement of nasogastric tubes or gastrostomies, irrigation, and patency assessment before nutrient administration. Pressure injury dressing changes involved selecting appropriate dressings based on the injury stage, with procedures like debridement, irrigation, and wound protection determined based on individual nurse experience. Some cases also involved more complex procedures such as diabetic foot wound care.

#### 2.3.2 Experimental group

In response to multiple issues identified in the control group’s nursing services—including inconsistent operational standards, weak risk identification capabilities, delayed emergency response, and lack of standardized and informatized support for service processes—this study developed quality improvement strategies and implementation plans for “Internet + Nursing Services” based on HFMEA. This aimed to construct a systematic, closed-loop quality management system for “Internet + Nursing Services” to effectively enhance service standardization, safety, and patient satisfaction.

##### 2.3.2.1 Strategy development

**Figure 1:**
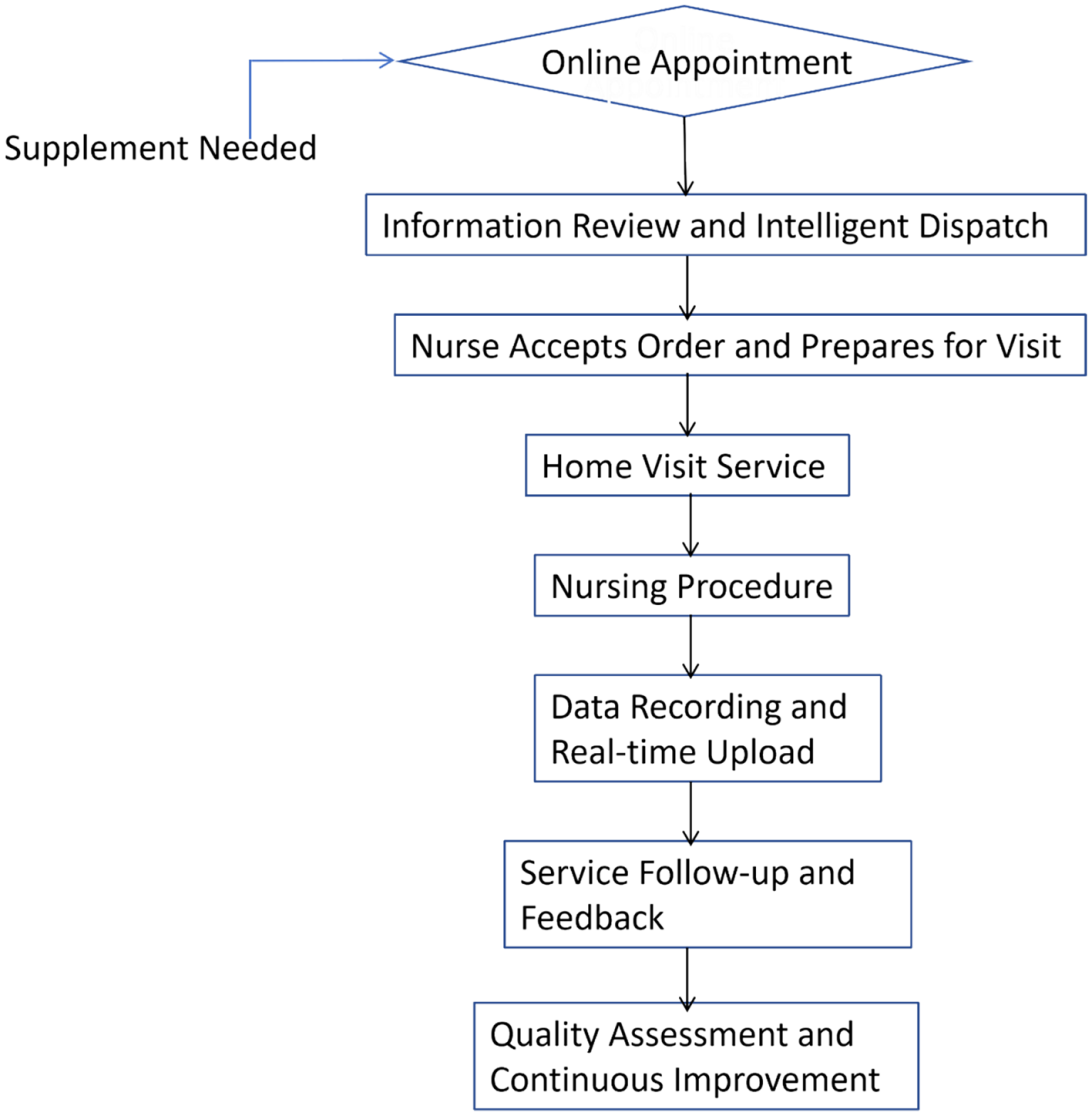
Standard workflow for “Internet + Nursing Services”.

First, a multidisciplinary quality assessment team was established, comprising the Director of Nursing, senior clinical nurses, information management personnel, and quality control experts. Clear division of responsibilities ensured comprehensive risk identification across all links from clinical practice to system management. Subsequently, the complete standard workflow for “Internet + Nursing Services” was delineated and mapped (see Figure 1), serving as the foundation for risk identification. Through flowchart analysis and brainstorming sessions, multiple potential failure modes were identified. These encompassed mismatches between nurse qualifications and assigned tasks during dispatch; patient information errors and incomplete material preparation pre-visit; catheter dislodgement, wound assessment errors, and inappropriate dressing selection during nursing procedures; improper symptom judgment and delayed response during emergencies; and data omission and lack of traceability during the recording and feedback stage. This provided a basis for formulating subsequent improvement measures.

After identifying potential failure modes in key links, a quantitative risk assessment was performed for each failure mode following the HFMEA methodology. The risk assessment employed a three-dimensional scoring principle: Severity (S): The consequence of the failure on patient health or service quality if it occurs, scored 1–10 (1: extremely minor impact, 10: extremely severe, potentially life-threatening). Occurrence (O): The likelihood of the failure mode occurring in the actual service process, scored 1–10 (1: extremely rare, 10: extremely frequent). Detection (D): The ease with which the failure is detected and corrected by the system before causing harm, scored 1–10 (1: extremely easy to detect and correct promptly, 10: extremely difficult to detect). The three scores were multiplied to obtain the Risk Priority Number (RPN) for each failure mode, calculated as: RPN = S × O × D.

RPN values were calculated and ranked for all identified failure modes, with a higher value indicating greater comprehensive risk for that link. Based on the assessment criteria, this study set an RPN ≥ 80 as the high-risk intervention threshold. Failure modes meeting or exceeding this value were prioritized for specific improvement measures and incorporated into key quality control and rectification plans.

##### 2.3.2.2 Nursing service

Patients in the experimental group utilized the “Internet + Nursing Service” platform co-developed by the hospital and a third-party WeChat platform. The process complied with “Internet + Nursing” requirements and interfaced with the hospital’s Hospital Information System (HIS), enabling functions such as online appointment, intelligent task dispatch, service evaluation, data tracking, and quality monitoring. The system automatically matched patients with nurses possessing appropriate qualifications based on the scheduled service type, prioritizing dispatch to nurses whose profile indicated specialization in the relevant nursing area. Nursing administrators pre-reviewed basic patient information, medical summaries, and nursing needs before service and conducted phone confirmation when necessary to ensure information accuracy.

All service nurses strictly adhered to the uniformly developed Standard Operating Procedures (SOPs). Key procedural steps incorporated checklists to ensure safety and standardization. For example, urethral catheter care followed aseptic technique, specified criteria for catheter replacement, and included perineal hygiene; nasogastric tube care verified tube fixation and assessed patency before infusion; pressure injury dressing changes selected appropriate dressings based on the injury stage, followed moist wound healing principles, and included dynamic efficacy evaluation. All nurses were equipped with standardized home-visit kits containing basic nursing supplies, assessment tools, infection prevention items, emergency medications and supplies, and patient health education materials.

All participating nurses were required to meet qualification criteria: being registered practicing nurses with over 5 years of clinical nursing experience, holding a professional title of Nurse Practitioner or higher, and having completed and passed specialized “Internet + Nursing Service” training and assessment. Training content included HFMEA fundamentals, risk identification, 31 nursing technical procedures, body-worn camera operation, process optimization, informatized data entry, emergency response, service etiquette, and communication skills. Performance evaluation was allowed only after passing the assessment. A performance management system incorporated service quality, patient satisfaction, adverse event reporting rates, and nursing record completion rates into assessment indicators to ensure continuous service improvement.

High-risk patients (e.g., long-term bedridden, severe underlying diseases, elderly living alone) were tagged with priority labels, triggering automatic system alerts for nursing staff. Nurses were also equipped with body-worn video cameras for emergency communication, enabling one-touch connection to platform physicians or emergency centers in case of emergencies. Regarding adverse event management, a “Nursing Adverse Event Reporting System” was established, requiring timely reporting, feedback, and rectification according to event severity levels, thereby forming a closed-loop management process. Furthermore, upon service completion, the platform automatically pushed satisfaction survey questionnaires covering aspects like communication attitude, technical skill, service responsiveness, and operational standardization, used for dynamic collection of patient feedback. The platform backend regularly generated quality data reports to analyze trending issues within services. The nursing quality control team organized quarterly quality review meetings, integrating HFMEA risk analysis results to continuously optimize service processes, promoting the standardized, sustainable, and safe development of “Internet + Nursing Services.”

### 2.4 Outcome measures

#### 2.4.1 Primary endpoints

- *Incidence of adverse events*: The proportion of clinically relevant nursing adverse events occurring during the service process, such as catheter dislodgement or delayed emergency response. Calculated as the percentage of the number of events divided by the total number of service encounters.
- *Rate of unplanned re-visits or hospital/clinic visits*: The occurrence rate of patients requiring another home visit or presenting to a healthcare facility within 48 h after receiving the service due to nursing-related issues or complications.
- *Mean Risk Priority Number (RPN) value (pre- vs. post-intervention comparison)*: Based on the HFMEA scoring system, the Severity (S), Occurrence (O), and Detection (D) scores were assigned to identified high-risk nursing process nodes before and after the intervention. The mean RPN was calculated to assess the effectiveness of risk control.

#### 2.4.2 Secondary endpoints

- *Patient satisfaction score*: Assessed using the platform’s built-in structured questionnaire, quantified on a 100-point scale. It covered five dimensions: service attitude, operational standardization, response timeliness, communication skills, and overall service experience. Each dimension had a maximum score of 20 points. The questionnaire demonstrated good internal consistency (Cronbach’s *α* = 0.87) and construct validity confirmed via confirmatory factor analysis (CFI = 0.95, RMSEA = 0.06), supporting its reliability for measuring patient satisfaction. The total score was the sum of the five-dimension scores, and the mean was used for analysis.
- *Nurse satisfaction score*: Similarly assessed using a platform-based 100-point quantitative evaluation. Evaluation dimensions included clarity of service processes, platform functional support, risk management safeguards, training effectiveness, and career development support. The scale showed good psychometric properties (Cronbach’s α = 0.85) and validated construct structure (CFI = 0.94, RMSEA = 0.07), ensuring robust measurement of nurse satisfaction. Each dimension had a maximum of 20 points. The total score was the sum of the five-dimension scores. This, combined with nurses’ subjective feedback, was used to comprehensively evaluate the feasibility of the implementation plan and nurse acceptance.

#### 2.4.3 Mechanism analysis

- *Mediation pathway analysis*: To elucidate the underlying mechanism, the hypothesized pathway where “specialized nurse training” influences “patient satisfaction” through the enhancement of the mediating variable “nurse risk perception capability” was tested using Structural Equation Modeling (SEM). The measurement model included validated scales for each latent construct, and the structural model specified the direct and indirect paths.

#### 2.4.4 Multidimensional performance

- *Six-dimensional quality profile*: Six core quality dimensions were selected for a comprehensive performance assessment: (1) Adverse Event Incidence, (2) Patient Satisfaction Score, (3) Nurse Satisfaction Score, (4) Nursing Record Completion Rate, (5) Response Timeliness (operationalized as the mean time from emergency alert to nurse acknowledgment), and (6) Process Efficiency (represented by the standardized mean operation time for core procedures, e.g., catheter care). For comparative visualization, raw scores for the experimental and control groups on each indicator were normalized to a 0–100 point scale using the formula: Standardized Score = (1 − (Actual Value − Minimum Value) / (Maximum Value − Minimum Value)) × 100. For positive indicators like satisfaction and completion rate, the formula was applied directly; for negative indicators like adverse event rate and response time, the ‘1 −’ component ensured higher standardized scores represented better performance.

#### 2.4.5 Adverse event structural indicators

- *Categorical distribution of adverse events*: To analyze changes in the pattern of adverse events, all recorded events were classified into five mutually exclusive categories: (1) Catheter-related events (e.g., unplanned extubation, mucosal injury), (2) Wound care events (e.g., inappropriate dressing selection, assessment error), (3) Material preparation events (e.g., wrong or missing supplies), (4) Emergency response events (e.g., delayed recognition or management of complications like vomiting post-feeding), and (5) Documentation/Communication events (e.g., missing key records, miscommunication). The proportional distribution (%) of each event type within the total events for each group was calculated and compared.

#### 2.4.6 Economic parameters

- *Direct medical cost analysis*: A preliminary cost-consequence analysis was conducted from the healthcare provider perspective. Direct medical costs associated with managing adverse events and unplanned re-visits were calculated for both groups. Cost categories included: (1) Cost of repeat home-visit services (based on standard service fee), (2) Cost of emergency department visits (including registration and basic treatment fees), (3) Cost of additional medications and consumables consumed due to adverse events or complications, and (4) Cost of additional nurse labor hours required for managing these events (calculated based on average hourly wage and documented extra time). Total cost per group, the absolute cost-saving amount for the experimental group, the cost-saving proportion, and the average cost-saving per service case (Total saving / number of service cases in experimental group) were derived.

### 2.5 Data collection

Data were extracted from the integrated “Internet + Nursing Service” platform database and the hospital’s quality management system. Specific data collection instruments included:

- *Nursing service electronic record*: A structured form completed by the nurse via the mobile platform immediately after each home visit, documenting nursing procedures performed, supplies used, patient condition, and any immediate outcomes.
- *Adverse event reporting form*: A mandatory electronic form triggered for completion by the involved nurse or quality controller within 24 h of any incident meeting the adverse event definition. It included event type, description, severity, immediate actions, and root cause analysis (post-intervention).
- *Satisfaction questionnaires*: Two separate, anonymized, 5-point Likert-scale questionnaires (converted to a 100-point scale for analysis) for patients and nurses. They were automatically distributed via the platform 24–72 h post-service. Patient questionnaire covered 5 domains (20 items total); nurse questionnaire covered 5 domains (18 items total).
- *HFMEA scoring worksheets*: Semi-structured forms used by the HFMEA multidisciplinary team during quarterly review meetings. They documented the S, O, D scores and RPN calculations for each pre-defined process node, along with rationale.
- *Cost data logs*: Data on service fees, consumable usage, and nurse activity logs were extracted from the platform’s billing and logistics modules and cross-referenced with payroll data for labor cost calculation.

### 2.6 Statistical analysis

All statistical analyses were performed using IBM SPSS Statistics (Version 23.0) and Mplus (Version 8.3).

- *Descriptive statistics*: Categorical variables were summarized as counts and percentages (*n*, %). Continuous variables were assessed for normality using the Shapiro–Wilk test and summarized as mean ± standard deviation (Mean ± SD) for normally distributed data, or median and interquartile range (Median [IQR]) for non-normal data.
- *Between-Group comparisons (Primary & Secondary Outcomes)*: For baseline characteristics and outcome comparisons between the propensity-score-matched control and experimental groups: (a) Categorical variables were compared using the Chi-square (*χ*^2^) test or Fisher’s exact test, as appropriate. (b) Normally distributed continuous variables were compared using the independent samples *t*-test. (c) Non-normally distributed continuous variables were compared using the Mann–Whitney U test.
- *Structural equation modeling (SEM)*: The hypothesized mediation model was tested using SEM in Mplus with the maximum likelihood estimation method. Model fit was evaluated using the following criteria: *χ*^2^/df < 3, Comparative Fit Index (CFI) > 0.95, Tucker-Lewis Index (TLI) > 0.95, Root Mean Square Error of Approximation (RMSEA) < 0.08, and Standardized Root Mean Square Residual (SRMR) < 0.08. The significance of the direct, indirect (mediation), and total effects was tested. Bias-corrected bootstrap confidence intervals (95% CI) with 5,000 resamples were calculated for the indirect effect to assess mediation.
- *Analysis of adverse event structure*: The difference in the proportional distribution of adverse event categories between the control and experimental groups was compared using the Chi-square test for homogeneity of proportions.
- *Economic data analysis*: Due to the skewed distribution of cost data, between-group comparisons of total costs and sub-category costs were performed using the Mann–Whitney U test. Descriptive statistics were used to present cost-saving amounts and proportions.
- *Standardization for radar chart*: The normalization of the six quality dimensions for radar chart presentation was performed using the predefined formula in SPSS. No inferential statistics were applied to these standardized scores; they were used solely for visual comparison.
- *Significance level*: A two-tailed *p*-value of less than 0.05 (*p* < 0.05) was considered statistically significant for all inferential tests.

## 3 Results

### 3.1 Comparative analysis of baseline characteristics

According to the inclusion and exclusion criteria, this study initially enrolled 170 patients in the control group and 400 patients in the experimental group. Comparison of baseline characteristics revealed no statistically significant differences between the two groups in terms of gender, age, underlying diseases (diabetes, stroke, other debilitated/long-term bedridden conditions), or primary nursing service items (urethral catheter care, nasogastric tube care, pressure injury dressing change) (all *p* > 0.05), as detailed in Table 1. This indicates comparability between the groups prior to the intervention.

### 3.2 Impact of HFMEA intervention on safety and adverse event profiles

#### 3.2.1 Incidence and structural shift of adverse events

During the study period, a total of 17 nursing-related adverse events were recorded, including unplanned extubation of nasogastric tubes, catheter-related injuries, supply errors, nasogastric feeding-associated complications, occupational exposure, and skin injuries. The overall incidence of adverse events was significantly lower in the experimental group (1.50%, 6/400) compared to the control group (6.47%, 11/170) (*χ*^2^ = 8.463, *p* = 0.003), representing a 76.8% relative reduction, with a risk difference of −4.97% (95% CI: −8.2 to −1.7%). Bonferroni correction was applied for multiple comparisons across adverse event types.

**Figure 2:**
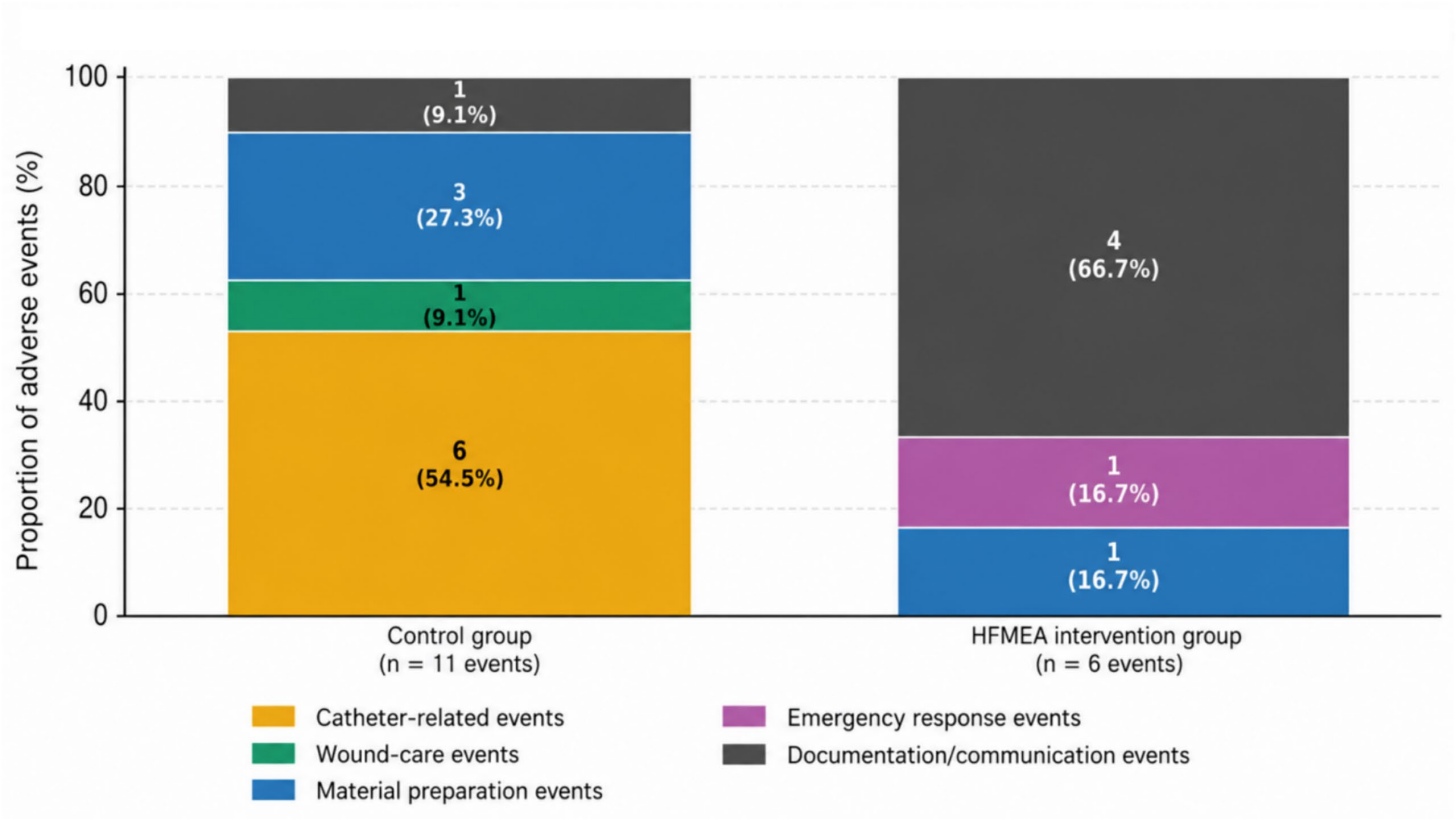
Distribution of adverse event types before and after HFMEA-based intervention.

To further elucidate the impact of the HFMEA intervention on process-level risks, all adverse events were categorized into five mutually exclusive types: (1) Catheter-related events, (2) Wound care events, (3) Material preparation events, (4) Emergency response events, and (5) Documentation/Communication events. Comparative analysis of proportional distribution revealed a structural shift post-intervention (Figure 2). In the control group, events were predominantly clustered in procedural and preparatory categories (Catheter-related, Wound care, Material preparation). Following the HFMEA-based intervention, absolute numbers in these categories drastically decreased, while the relative proportion of Documentation/Communication events increased.

#### 3.2.2 Rate of unplanned re-visits or hospital/clinic visits

Within 48 h post-service, 18 patients (10.6%) in the control group required an unplanned repeat home visit or presented to a healthcare facility due to reasons such as incomplete care, omitted supplies, or inadequate wound management. In contrast, only 9 patients (2.25%) in the experimental group experienced such events (*χ*^2^ = 18.38, *p* < 0.001), representing a relative risk reduction of 78.8% and a risk difference of −8.35% (95% CI: −12.4% to −4.3%). Bonferroni correction was applied to adjust for multiple outcome comparisons.

This finding underscores a substantial improvement in the effectiveness and completeness of nursing care delivered under the HFMEA-optimized model. The observed reductions align with the concurrent declines in mean RPN scores across high-risk nodes (Supplementary Table S1), reflecting the impact of targeted interventions—such as automated nurse–patient matching, pre-visit checklists, and standardized operating procedures—on mitigating latent process risks.

#### 3.2.3 Summary: mean RPN reduction

The aggregate mean RPN score, reflecting composite risk (Severity × Occurrence × Detection), decreased from 172.1 ± 20.8 in the control group to 34.6 ± 10.2 post-intervention, representing an approximate 80% reduction. This substantial decrease confirms that the HFMEA-based interventions effectively mitigated latent process risks across the INS workflow, aligning with observed improvements in adverse event incidence and unplanned revisit rates.

### 3.3 Enhancement of stakeholder experience and operational metrics

#### 3.3.1 Patient satisfaction

Valid patient satisfaction questionnaires were collected from 546 respondents (158 control, 388 experimental). The mean overall satisfaction score was significantly higher in the experimental group (97.2 ± 2.1) compared to the control group (92.0 ± 2.7) (*p* < 0.001). Superior scores in the experimental group were consistent across all evaluated dimensions: operational standardization, service attitude, response timeliness, communication skills, satisfaction with health guidance, and overall service experience (all *p* < 0.001), as detailed in Table 2.

**Table 2:** Comparison of patient satisfaction scores across dimensions (mean ± SD).

Dimension	Control group (*n* = 158)	Experimental group (*n* = 388)	*t*-value	*p*-value
Operational standardization	91.5 ± 3.0	97.5 ± 2.0	20.13	<0.001
Service attitude	92.4 ± 2.5	97.8 ± 1.8	21.27	<0.001
Response timeliness	91.8 ± 2.8	97.1 ± 2.2	19.88	<0.001
Communication skills	91.9 ± 2.7	97.0 ± 2.3	19.54	<0.001
Satisfaction with health guidance	91.3 ± 3.1	96.8 ± 2.4	18.92	<0.001
Overall service experience	92.6 ± 2.4	97.6 ± 1.9	21.65	<0.001
Average total score	92.0 ± 2.7	97.2 ± 2.1	2	

#### 3.3.2 Nurse satisfaction

Satisfaction scores from the 42 registered nurses were analyzed separately for the pre-intervention (2022) and post-intervention (2023) periods. The mean overall nurse satisfaction score increased significantly from 77.0 ± 13.8 pre-intervention to 90.6 ± 8.4 post-intervention (*p* < 0.001). Marked improvements were observed in key dimensions such as process clarity, risk controllability, and platform support (all *p* < 0.001), indicating enhanced work experience and professional confidence under the new model (Table 3).

**Table 3:** Comparison of nurse satisfaction scores across two phases (*n* = 42).

Dimension	Jan 1, 2022 – Dec 31, 2022 (mean ± SD)	Jan 1, 2023 – Dec 31, 2023 (mean ± SD)	*t*-value	*p*-value
Process clarity	76.0 ± 13.2	91.0 ± 8.0	5.46	<0.001
Risk controllability	74.4 ± 14.0	90.0 ± 8.4	5.12	<0.001
Platform support	78.0 ± 12.0	92.4 ± 7.0	5.83	<0.001
Reduced burnout	84.0 ± 10.0	92.0 ± 7.5	2.99	0.005
Service satisfaction	86.0 ± 9.0	94.0 ± 6.5	3.29	0.003
Sense of accomplishment	86.4 ± 9.6	94.6 ± 6.2	3.27	0.003
Average total score	77.0 ± 13.8	90.6 ± 8.4		

#### 3.3.3 Nursing record completion and response timeliness

The experimental group’s implementation of structured electronic templates coupled with automated platform reminders resulted in a nursing record completion rate of 98.2%, markedly superior to the 85.9% rate in the control group. Furthermore, the average response time to emergent situations was reduced from 42 min in the control group to 21 min in the experimental group, representing a 50% improvement in response agility.

### 3.4 Mechanistic insights: mediating role of risk perception

#### 3.4.1 Structural equation modeling (SEM) analysis

**Figure 3:**
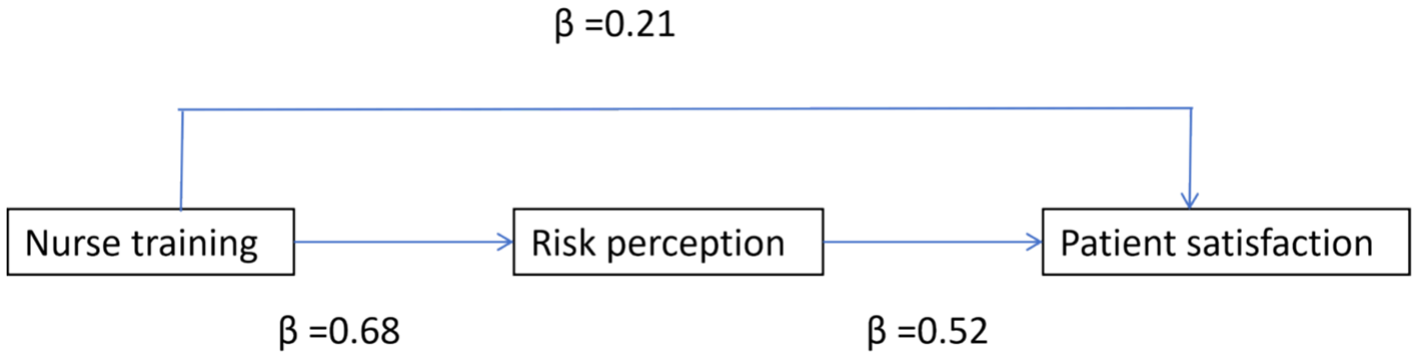
Structural equation model path diagram: Nurse training influencing patient satisfaction through risk perception as a mediator.

To unravel the underlying mechanism through which the HFMEA intervention influences patient satisfaction, a Structural Equation Model was constructed. The model hypothesized that “specialized nurse training” enhances “patient satisfaction” indirectly by strengthening the mediating variable “nurse risk perception capability.” The model demonstrated excellent fit (*χ*^2^/df = 2.15, CFI = 0.96, TLI = 0.94, RMSEA = 0.06, SRMR = 0.04). Path analysis (Table 4) confirmed significant direct and indirect effects. Mediation analysis revealed that risk perception played a substantial partial mediating role, with an indirect effect value of 0.35 (95% CI: 0.24–0.46), accounting for 62.5% of the total effect. (Figure 3) This elucidates that the intervention’s success is mechanistically driven not only by procedural standardization but also by enhancing nurses’ cognitive capacity for risk identification and management, which in turn improves patient experience.

**Table 4:** Path coefficients and significance testing in the structural equation model.

Path	Standardized coefficient (β)	Standard error (SE)	*Z*-value	*p*-value
Nurse training → risk perception	0.68	0.07	9.71	<0.001
Risk perception → patient satisfaction	0.52	0.08	6.5	<0.001
Nurse training → patient satisfaction (direct)	0.21	0.06	3.5	<0.001
Nurse training → patient satisfaction (total)	0.56	0.08		

The mediation analysis indicated that risk perception played a partial mediating role between nurse training and patient satisfaction. The indirect effect was 0.35 (95% CI: 0.24–0.46), accounting for 62.5% of the total effect. This result elucidates, at a mechanistic level, that the HFMEA intervention not only directly improved service quality through process standardization but also indirectly enhanced patient experience by strengthening nurses’ capabilities in risk identification and management.

### 3.5 Multidimensional quality performance and economic evaluation

#### 3.5.1 Holistic quality assessment via six-dimensional radar chart

**Figure 4:**
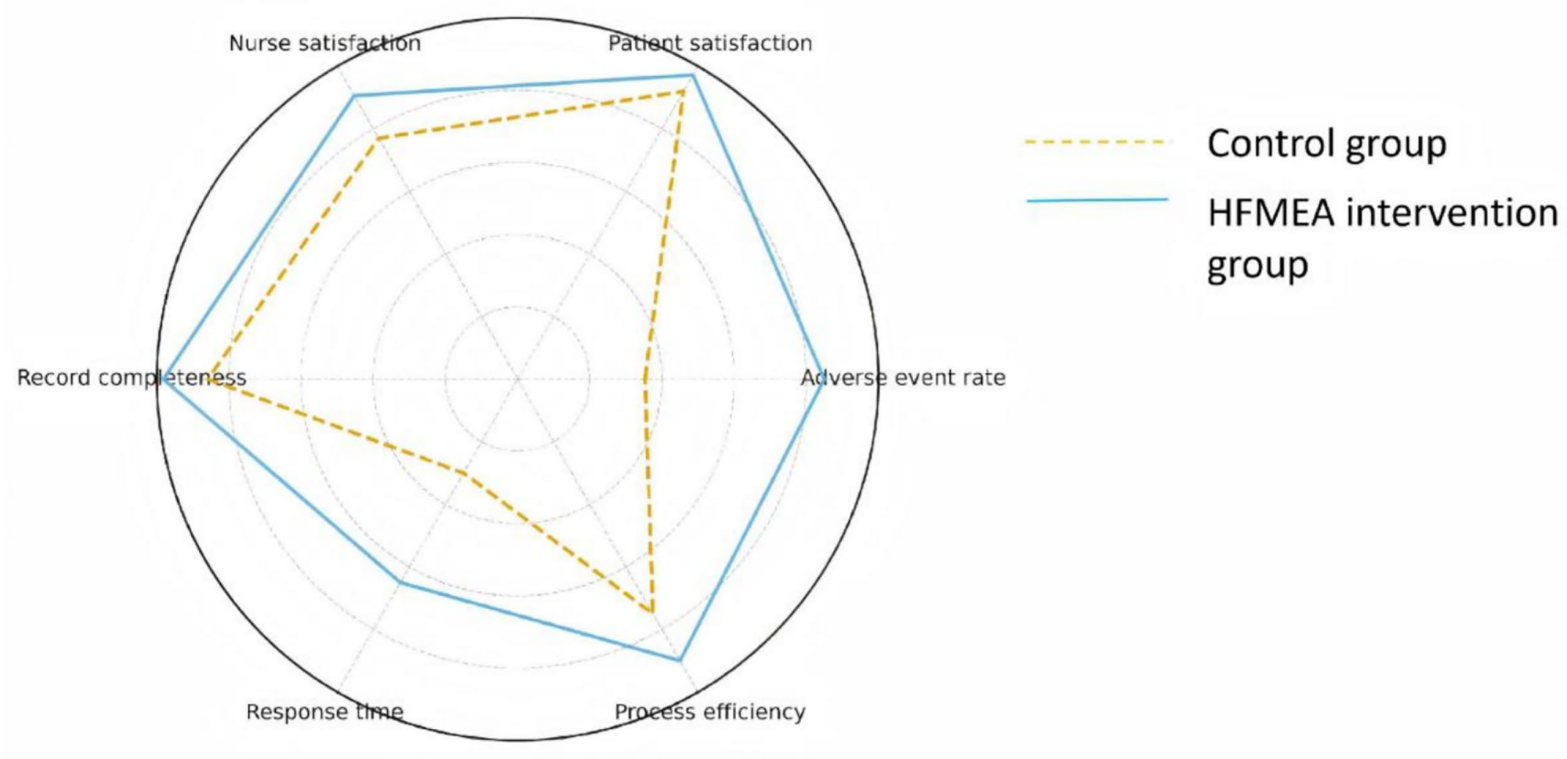
Six-dimensional radar chart of nursing quality (solid line: experimental group; dashed line: control group).

A composite evaluation was conducted across six core quality dimensions: Adverse Event Incidence, Patient Satisfaction, Nurse Satisfaction, Nursing Record Completion Rate, Response Timeliness, and Process Efficiency (represented by standardized mean operation time). After normalization to a 0–100 scale for visual comparison, the radar chart (Figure 4) clearly demonstrates that the experimental group outperformed the control group across all six dimensions. The most pronounced advancements were observed in safety (Adverse Event Control) and efficiency metrics (Response Timeliness, Process Efficiency), resulting in a more balanced and robust quality profile for the HFMEA-optimized service model.

#### 3.5.2 Preliminary cost-consequence analysis

A preliminary analysis of direct medical costs associated with adverse event management and unplanned re-visits was conducted from the healthcare provider perspective. As shown in Table 5, the total cost was significantly lower in the experimental group across all cost categories (repeat home-visit service, emergency department visits, additional medications/consumables, extra nursing labor hours). The HFMEA intervention required initial investments, including development and integration of the digital platform (~50,000 RMB) and procurement of wearable devices for emergency communication (~12,000 RMB). Despite these upfront costs, the intervention yielded a total cost saving of 16,530 RMB, corresponding to a 75.6% reduction relative to the control group, with an average of 41.3 RMB saved per service case. Preliminary estimates of potential ROI suggest that for a scaling to 1,000 service cases per year, savings could exceed 41,000 RMB annually, supporting both cost-effectiveness and feasibility for replication in similar institutional settings, though actual ROI will depend on local labor, technology, and operational costs. These findings suggest that the HFMEA-based quality management model not only enhances care quality and safety but also demonstrates considerable potential for economic savings, indicating favorable cost-effectiveness.

**Table 5:** Comparison of direct medical costs related to adverse events between groups (RMB).

Cost category	Control group (*n* = 170)	Experimental group (*n* = 400)	Cost saving	Saving rate
Repeat home-visit service cost	8,520	2,250	6,270	73.60%
Emergency department visit cost	5,670	1,080	4,590	81.00%
Additional consumables cost	3,450	890	2,560	74.20%
Additional nurse labor hour cost	4,230	1,120	3,110	73.50%
Total	21,870	5,340	16,530	75.60%

The experimental group incurred significantly lower direct medical costs related to adverse events compared to the control group. Calculated per service case, the average cost saving was approximately 41.3 RMB.

Initial investments included digital platform development (~50,000 RMB) and wearable devices for nurses (~12,000 RMB).

Preliminary estimates suggest that scaling to 1,000 service cases/year could yield savings >41,000 RMB, indicating favorable ROI and potential for replication in similar healthcare settings.

## 4 Discussion

This study applied HFMEA as a prospective quality management tool to systematically evaluate and restructure the workflow of INS. The intervention—comprising workflow standardization, structured nurse training, and digital platform integration—was associated with improvements in nursing quality and safety, including enhanced risk identification, process compliance, and emergency response (15, 16). However, given the retrospective, single-center design and the use of historical controls (2022 vs. 2023), the observed associations should not be interpreted as definitive causal effects. Multiple contextual, temporal, and organizational factors may have contributed to the outcomes, and these are discussed below.

The study found a 76.8% relative reduction in adverse events, an 80% decrease in mean RPN scores, and meaningful gains in stakeholder satisfaction. While these findings are promising, they must be interpreted with caution. The intervention period (2023) coincided with ongoing organizational learning, potential secular improvements in INS management, and possible heightened awareness among nurses due to the study itself (Hawthorne effect). Moreover, the use of propensity score matching cannot eliminate all unmeasured confounding, such as differences in patient health literacy, caregiver support, or home environmental risks that may have varied between the two time periods. Therefore, we describe these results as associations rather than direct effects of the HFMEA intervention alone.

The substantial decline in adverse event incidence aligns with the preventive philosophy of HFMEA, which emphasizes identifying, assessing, and strategically mitigating potential failure modes within critical process nodes (9). In our intervention, error-proofing mechanisms were implemented across key nodes—dispatch, pre-visit preparation, nursing procedures, and documentation—including automatic nurse qualification matching, pre-visit checklist verification, dissemination of standard operating manuals, emergency procedure drills, and electronic record reminders. These measures likely reduced adverse events caused by human variability and process inconsistencies, consistent with the concept of “forward identification” in home care risk management described by Yoshimatsu et al. (17).

The pronounced post-intervention reduction in mean RPN values suggests that systematic identification of high-risk process nodes, coupled with nurse-led implementation of HFMEA controls, may effectively mitigate latent risks (18). Nurses applied interventions such as automated nurse–patient matching, pre-visit verification, and structured documentation to reduce variability and enhance proactive safety management (19). Our HFMEA team systematically identified failure modes and performed RPN scoring for eight key links in the INS workflow. The control group’s mean RPN was 172.1 ± 20.8, with “Nursing Operations” (RPN = 270) and “Inadequate Pre-visit Preparation” (RPN = 225) posing the highest risks (20). Post-intervention, the experimental group’s mean RPN dropped sharply to 34.6 ± 10.2, representing a reduction exceeding 80% for high-risk links. This trend aligns with the observed declines in adverse events and unplanned revisit rates, suggesting that HFMEA was associated with reduced latent process risks. This finding is consistent with research by Shekelle et al. (21) on the value of protocol standardization, and mirrors results reported by Moradi et al. (22), who achieved over 40% RPN reduction applying HFMEA in emergency department nursing processes.

The 50% reduction in emergency response time and the marked decrease in unplanned re-visits suggest meaningful improvements in care process efficiency. These align with research by Bates and Singh (23) on how structured approaches can streamline processes. The integration of digital tools with standardized workflows may have created synergistic effects, though the observational design precludes attributing causality to any single component.

A noteworthy observation is the structural shift in reported adverse event types. While the control group experienced primarily procedure-related incidents, the experimental group showed a relative increase in documentation and communication issues within the total event pool. This pattern, also described by Pronovost et al. (24) in other settings, likely reflects improved detection systems and a stronger reporting culture that made previously undetected or unreported issues visible, rather than a deterioration in documentation quality. Our near-perfect nursing record completion rate (98.2%) supports this interpretation, indicating enhanced system sensitivity and transparency—a hallmark of a mature safety culture (20). Nevertheless, it is possible that some documentation issues became more visible simply because the digital platform made them easier to track; future research should distinguish between true changes in event occurrence versus changes in detection.

Regarding service experience, patient satisfaction scores improved across multiple dimensions including “operational standardization,” “service attitude,” “response timeliness,” “communication skills,” and “health guidance,” with the overall mean score increasing from 92.0 to 97.2. This aligns with established correlations between patient satisfaction and clinical outcomes (25). The HFMEA intervention promoted digitization, structuring, and standardization of service processes, which likely enhanced predictability and consistency. For instance, the platform-enabled automatic dispatch and qualification matching allowed nurses to access complete patient information before home visits, while post-service automated satisfaction surveys integrated patient feedback into a closed-loop quality improvement cycle. This resonates with findings by Ren et al. in INS for dysphagia care, where technological empowerment optimized processes, increased transparency, and bolstered patient trust and satisfaction (26). Furthermore, standardized training for the experimental group nurses ensured consistent operational standards. However, satisfaction scores were collected via self-report, raising the possibility of social desirability bias, especially among nurses who participated in the intervention.

Nurse satisfaction also improved, with higher scores in the experimental group for “process clarity,” “risk controllability,” and “platform support.” This suggests that with standardized processes and system support, nurses gained clearer understanding of their responsibilities and risk expectations, potentially alleviating practice pressure and enhancing professional confidence and fulfillment. This is crucial given the established link between nurse satisfaction, care quality, and patient safety (27). Zhang et al. (28) also noted that in INS practice, a lack of clear process guidance and systematic training can lead to competency mismatch and role anxiety, increasing psychological burden and potentially affecting staff retention. In contrast, HFMEA as a structured process risk intervention tool may have contributed not only to service quality but also to providing nurses with a clearer, more controlled work environment and a channel for professional growth.

The mediation analysis revealing that nurse risk perception accounts for 62.5% of the total effect on patient satisfaction provides exploratory insights into how structural interventions might influence experiential outcomes. This supports cognitive theories of clinical decision-making (29). The HFMEA process, emphasizing systematic risk identification, may have enhanced nurses’ situational awareness and anticipatory thinking, aligning with Croskerry’s (30) work on mindful practice. This cognitive shift offers one plausible explanation for the effectiveness of checklists and protocols, though causal mediation would require longitudinal or experimental designs.

The cost analysis showing savings of 16,530 RMB suggests potential economic benefits, but several caveats apply. The analysis included only direct costs associated with adverse event management and unplanned re-visits, excluding upstream implementation costs such as platform development and wearable devices. Additionally, cost estimates were based on a single institution’s pricing and labor rates, which may not generalize to other settings. Indirect costs (e.g., patient time, caregiver burden) were not captured. Therefore, while these preliminary findings align with research on capturing downstream cost effects (31), we caution against overgeneralizing the economic conclusions. Formal cost-effectiveness analyses with longer time horizons are needed.

Beyond the limitations already mentioned, several contextual and organizational factors warrant deeper reflection. The model’s success in our setting relied on enabling conditions including supportive hospital administration, existing digital infrastructure, a dedicated multidisciplinary team, and a relatively homogeneous local regulatory environment. Key barriers to implementation in other digital healthcare settings include: (1) technological barriers (interoperability between different information systems); (2) organizational barriers (need for trained facilitators, protected meeting time, and a proactive safety culture); (3) regulatory and legal barriers (data privacy, cross-regional licensure, liability for home visit adverse events); and (4) financial sustainability (initial investment costs and variable reimbursement policies). These factors may considerably affect the transferability and sustainability of the HFMEA-based model in different institutional contexts.

Several limitations should be considered. The single-center design affects generalizability, though the detailed methodology may aid adaptation. The one-year follow-up period limits assessment of long-term sustainability. While propensity score matching controlled for many confounders, unmeasured variables (e.g., caregiver competence, home environment hazards, seasonal variations) may have influenced results. Additionally, the satisfaction questionnaires, while showing acceptable internal consistency in this study, have not been externally validated. The low absolute number of adverse events in the experimental group (n = 6) limits statistical precision for category-specific comparisons.

Future research should involve multicenter prospective studies with longer follow-up, ideally using interrupted time series or stepped-wedge designs to better control for temporal trends. Investigating which HFMEA components contribute most to outcomes could optimize resource allocation. Studies on how digital tool design influences risk management effectiveness could inform technology development for home care services. Implementation science frameworks (e.g., RE-AIM, CFIR) could be used to systematically assess barriers and facilitators when adapting the model to diverse healthcare systems.

## 5 Conclusion

This study suggests that the systematic application of HFMEA methodology is associated with improvements in safety, efficiency, and stakeholder satisfaction within the context of “Internet + Nursing Services.” By integrating structured risk assessment, digital workflow management, and targeted training, the HFMEA-based model may contribute to enhanced reliability and quality of home nursing care. The observed reductions in adverse events and process risk scores, along with increased patient and nurse satisfaction, are promising; however, given the retrospective, single-center design and the lack of long-term follow-up, these findings should be interpreted as associations rather than definitive causal effects. The preliminary economic analysis indicates potential cost savings, but these results are not yet generalizable, and formal cost-effectiveness studies are needed. Replication of this model in other healthcare settings would likely require adaptation to local regulatory frameworks, resource availability, digital infrastructure, and organizational culture, and its sustainability remains to be evaluated. In summary, while the HFMEA-based quality management model shows practical and innovative potential, future prospective, multicenter research with extended follow-up is necessary to confirm its effectiveness and transferability. As digital health services continue to expand, robust quality management frameworks remain essential, and this study offers a cautious, evidence-informed reference for implementing technology-enabled home care.

## Data Availability

The raw data supporting the conclusions of this article will be made available by the authors, without undue reservation.
