# Supplementary material for: Construction of a safe and controllable quality management model for “Internet + Nursing Services” based on HFMEA strategy
Source: Front Med (Lausanne). 2026 Jun 3;13:1797741. doi: 10.3389/fmed.2026.1797741 (PMC13272465; doi:10.3389/fmed.2026.1797741)
Supplement: Supplementary file 1 [file Table_1.docx]

Table S1. Risk Priority Number (RPN) for Key Process Nodes Before and After HFMEA-Based Intervention

| **Process Node** | **RPN (Control)** | **RPN (Experimental)** | **Reduction (%)** | **Key Intervention** |
| --- | --- | --- | --- | --- |
| Nursing Operations | 270 | 35 | 87 | SOP implementation, automated nurse-patient matching, standardized task assignment |
| Pre-visit Preparation | 225 | 30 | 86.7 | Pre-visit checklist, supply verification, patient information confirmation |
| Catheter Care | 180 | 25 | 86.1 | Standardized catheter procedures, nurse training, aseptic technique adherence |
| Wound Dressing | 160 | 28 | 82.5 | Wound care SOP, dressing selection guidelines, structured nurse training |
| Nasogastric Tube Care | 155 | 27 | 82.6 | Tube care SOP, pre-infusion patency check, nurse competency verification |
| Emergency Response | 145 | 30 | 79.3 | Emergency drills, SOPs for complications, wearable camera for rapid communication |
| Material Preparation | 130 | 25 | 80.8 | Standardized supply kits, pre-visit material checklists |
| Documentation/Feedback | 120 | 26 | 78.3 | Electronic record templates, automated reminders, closed-loop reporting system |
| **Mean ± SD** | 172.1 ± 20.8 | 34.6 ± 10.2 | ~80% | — |

Note: RPN values are calculated as Severity × Occurrence × Detection for each failure mode.

High-risk nodes (RPN ≥ 80) were prioritized for targeted interventions listed above.
